# NAFLD and Chronic Kidney Disease

**DOI:** 10.3390/ijms17040562

**Published:** 2016-04-14

**Authors:** Morgan Marcuccilli, Michel Chonchol

**Affiliations:** 1 Division of Renal Diseases and Hypertension, University of Colorado Hospital, Aurora, CO 80045, USA; 2 Division of Renal Diseases and Hypertension, University of Colorado Denver, 13199 East Montview Boulevard, Suite 495, Aurora, CO 80045, USA

**Keywords:** non-alcoholic fatty liver disease, chronic kidney disease, non-alcoholic steatohepatitis, inflammation, review

## Abstract

Non-alcoholic fatty liver disease (NAFLD) is the most common cause of chronic liver disease in developed countries and it is now considered a risk factor for cardiovascular disease. Evidence linking NAFLD to the development and progression of chronic kidney disease (CKD) is emerging as a popular area of scientific interest. The rise in simultaneous liver-kidney transplantation as well as the significant cost associated with the presence of chronic kidney disease in the NAFLD population make this entity a worthwhile target for screening and therapeutic intervention. While several cross-sectional and case control studies have been published to substantiate these theories, very little data exists on the underlying cause of NAFLD and CKD. In this review, we will discuss the most recent publications on the diagnosis of NAFLD as well new evidence regarding the pathophysiology of NAFLD and CKD as an inflammatory disorder. These mechanisms include the role of obesity, the renin-angiotensin system, and dysregulation of fructose metabolism and lipogenesis in the development of both disorders. Further investigation of these pathways may lead to novel therapies that aim to target the NAFLD and CKD. However, more prospective studies that include information on both renal and liver histology will be necessary in order to understand the relationship between these diseases.

## 1. Introduction

Non-alcoholic fatty liver disease (NAFLD) is the most common cause of chronic liver disease worldwide [1]. It is defined as the accumulation of fat (>5%) in liver cells in the absence of excessive alcohol intake or other causes of liver disease including autoimmune, drug-induced, or viral hepatitis [2]. The histologic spectrum of NAFLD ranges from simple steatosis to non-alcoholic steatohepatitis (NASH), liver fibrosis, and cirrhosis [2]. This disease reportedly affects up to 30% of the general population in Western countries, especially in patients with metabolic syndrome, obesity, and type II diabetes [3]. Given the high prevalence of this disease, it has recently been associated with hepatocellular carcinoma (HCC) [3]. In addition, NASH as the primary indication for liver transplantation has increased from 1.2% to 9.7% in the last decade [3]. NAFLD is considered to be an independent risk factor for cardiovascular disease and there is accumulating evidence to support a causative role in the development of chronic kidney disease (CKD) [3].

In addition to NAFLD, CKD represents a significant health burden in the Western adult population, and it affects over 25% of individuals older than 65 years [4]. CKD is defined as decreased estimated glomerular filtration (eGFR) and/or the presence of significant proteinuria (>500 mg) [5]. In the United States, over 400,000 people currently receive some form of renal replacement therapy, and this number is expected to reach 2.2 million by 2030 [6]. However, less than half of CKD patients develop end stage renal disease due to the high risk of mortality associated with cardiovascular events [7]. Furthermore, the incidence of simultaneous liver-kidney transplantation continues to increase exponentially over the last five years [3]. An analysis of the United Network Organ Sharing (UNOS) database during the years 2002–2011, revealed that 35% of patients transplanted for NAFLD-related cirrhosis progressed to stage 3b-4 CKD within two years after liver transplantation in comparison to 10% of patients transplanted for other etiologies [8]. Despite these findings, CKD often goes unrecognized and in the Third National Health and Nutrition Survey (NHANES III), among all individuals with moderately decreased GFR (<60 mL/min; Stage 3), the awareness is approximately 8% [9].

The similarity in traditional risk factors for CKD including hypertension, obesity, dyslipidemia, and insulin resistance make it difficult to determine a causational relationship with NAFLD adjusting for “hepatorenal” and “cardiorenal” features [5]. While a multitude of cross-sectional and longitudinal studies exist, there is still very little prospective data linking NAFLD to CKD. In addition, underlying mechanisms related to inflammation, oxidative stress, and fibrogenesis are currently being investigated in the development of kidney injury in the presence of fatty liver disease [5]. In this review, we will examine new data on the diagnosis of NAFLD, current evidence linking NAFLD to CKD, and new studies revealing the underlying pathophysiology and potential treatments of these globally burdensome diseases.

## 2. Diagnosis and Screening

### 2.1. Imaging

Liver biopsy remains the gold standard of diagnosis for NAFLD or NASH. Histologic classifications range from simple steatosis to advanced periportal or perisinusoidal fibrosis [10]. However, a considerable proportion of patients are not diagnosed with NAFLD by biopsy, and this method is unreliable secondary to subjectivity of histologic interpretation as well as sample bias related to patchiness of its distribution in the liver [10]. Ultrasonography remains the recommended first-line imaging modality for diagnosing hepatic lipid accumulation in clinical practice. This method of screening is limited if >30% of hepatocytes are steatoic given its reliance of echogenicity or contrast [5]. A recent meta-analysis has shown that the overall sensitivity and specificity of ultrasonography for the detection of moderate to severe fatty liver compared to histology were 84.8% and 93.6% [11].

Other methods of diagnosis include magnetic resonance imaging (MRI), which can assess decreased liver signal intensity, and proton magnetic resonance spectroscopy, which is used for measuring the area under the lipid spectrum relative to water spectrum [12]. These diagnostic techniques are excellent for assessing the quantitative severity of liver fat accumulation, however, they cannot discriminate simple steatosis from lipid accumulation associated with inflammation and fibrosis (*i.e.*, NASH) [12]. According to systematic review, simple steatosis and NASH are considered different disease states each with its own pathogenesis and cardiovascular risk. In addition, it may be possible that NASH can occur in the absence of simple steatosis and the pathogenesis leading to the progression to fibrosis/cirrhosis is still not entirely clear [13]. Nevertheless, NASH is often progressive, with development of advanced fibrosis in 30%–40% of patients, cirrhosis in 15%–20%, and liver failure in 2%–4% [5].

Another modality for the assessment of NAFLD that has recently gained popularity is the use of transient elastography (TE; Fibroscan^®^, Echosens, Paris, France), which measures liver stiffness using an ultrasound probe [14]. A new physical parameter based on the properties of ultrasonic signals acquired by this machine has been recently developed to assess liver steatosis known as the controlled attenuation parameter (CAP) score. [14]. A recent study measured the CAP score on 62 patients with CKD stage III and IV in order to quantify liver steatosis and concluded that 53 patients had NAFLD with a positive correlation between severity of liver steatosis and serum creatinine (*p* < 0.01). Limitations included the cross-sectional format of this investigation, which does not allow conclusions to be causal, as well as the absence of a control group of non-steatotic patients, or confirmation of findings by liver biopsy in comparison to CAP score [14]. This study determined that that the severity of liver steatosis is negatively correlated with kidney function, and it documents the value of ultra-sonographic elastography as an effective non-invasive screening method for the diagnosis of NAFLD [14].

### 2.2. Liver Enzymes and Biomarkers

In addition to imaging, many investigators have explored the use of serum tests in NAFLD ideally for diagnosis, monitoring progression, response to therapeutic intervention, and determining the prognosis of the disease. Mildly elevated serum aminotransferase levels are the primary abnormality seen in patients with NAFLD, however, liver enzymes (LFTs) may be normal in up to 78% of patients with NAFLD [15]. A recent study published by Mikolasevic and associates examined the use of liver enzymes *versus* CAP score in the detection of NAFLD in patients with CKD and coronary artery disease (CAD). This was a cross-sectional study of 202 patients with CKD, end-stage renal disease (ESRD), renal transplant recipients (RTRs) and patients with proven CAD matched against individuals without elevated LFTs and normal kidney function [15]. According to the CAP findings, 76.9% of CKD patients, 82% ESRD patients, 74% RTRs, and 69.1% CAD patients had CAP > 238 decibels to milliwatt (dB.m) and thus by definition NAFLD. However, the results demonstrated that LFTs correlated with liver stiffness acquired with TE only in CAD patients, and therefore is not a reliable marker of the detection of NAFLD in patients with renal disease [15].

While several other biomarkers have been implicated in the diagnosis and screening of NAFLD, there is still a lack of reproducibility in their clinical application. Tumor necrosis factor (TNF-α), which plays an important role in insulin resistance through inhibition of the tyrosine kinase activity of the insulin receptor, has recently gained attention for its potential value [16]. One study reported that patients with NASH had significantly higher serum TNF-α than those with simple steatosis, while another recent study further stated that patients with NASH had higher levels of TNF-α messenger ribonucleic acid (mRNA) than healthy controls with a sensitivity 66.7% and a specificity 74.1% [16]. Still, there are no known studies reporting the relationship of TNF-α as a marker of both NAFLD and CKD. Other potential biomarkers include interleukin-6 (IL-6), adiponectin, and pentraxin-3 (PTX3) are also under investigation [16].

The development of panels has also shown promise in non-invasive testing for NAFLD. There are scoring systems available for the prediction of the presence NASH as well as for prognosis of advanced fibrosis (see Table 1) [17,18,19,20,21,22,23,24,25]. Diagnostic panels are thought to be more applicable for patients with a BMI > 35 and the presence of hypertension as well as age >50 years [26]. FIB-4 score is a prognostic panel composed of age, alanine aminotransferase (ALT), aspart aminotransferase (AST), and platelet count [27]. A recent study published in *Hepatology Intl.* compared these scoring systems in an effort to identify the presence of CKD in patients with NAFLD. A total of 755 patients diagnosed with NAFLD by ultrasound were assessed for glomerular filtration rate, AST to ALT ratio, AST to platelet ratio, FIB-4 score, NAFLD fibrosis score, and BARD score [27]. The results revealed that a cut-off value of 1.100 for FIB-4 score gave a sensitivity of 68.85% and a specificity of 71.07% for predicting CKD, and only the FIB-4 score, older age, higher uric acid level, and elevated diastolic blood pressure were independent predictors of CKD in comparison to the other scoring panels [27]. While this study was cross-sectional and limited by ultrasound diagnosis of NAFLD, the investigators concluded that a high noninvasive fibrosis score is associated with an increased risk of prevalent CKD, and that FIB-4 is the better predictor than other fibrosis scores in excluding the presence of CKD in patients with NAFLD [27]. Ideally, a combination of non-invasive imaging and serum biomarkers will be verified for practical application in the clinical detection of both NAFLD and CKD.

## 3. Epidemiologic Evidence Linking Chronic Kidney Disease (CKD) to Non-Alcoholic Fatty Liver Disease (NAFLD)

As stated above, the similarity in risk factors for NAFLD and CKD including obesity, diabetes, and hypertension make it difficult to delineate a direct association between the diagnosis of fatty liver disease and the development and progression of renal disease. A recent meta-analysis of thirty-three studies for a total of over two-thousand participants found that NAFLD was associated with an increased prevalence odd ratio (OR) 2.12, 95% confidence interval (CI), 1.69–2.66 as well as incidence hazard ratio (HR) 1.79, 95% CI 1.65–1.95 of CKD [28]. In Table 2, there several large cross-sectional as well as case control studies of patients with NAFLD showing the prevalence of CKD between 4%–40% (see Table 2) [29,30,31,32,33,34,35,36,37,38,39,40,41,42,43,44,45,46,47,48,49,50]. In addition, there appears to be a correlation between the severity of NAFLD and the progression of CKD [51]. However, nearly half of these studies use ultrasound for the diagnosis of NAFLD or NASH as opposed to biopsy [29,30,31,32,33,34,35,36,37,38,39,40,41,42,43,44,45,46,47,48,49,50]. Other limitations include the use of Modification of Diet in Renal Disease (MDRD) and the Chronic Kidney Disease Epidemiology Collaboration (CKD-EPI) algorithms to calculate eGFR, neither of which are reliable in the presence of obesity or cirrhosis [5]. There is also substantial variability in the patient groups studied in regards ethnicity, age, risk factors, and selection bias using hospital based cohorts that often represent a population with advanced disease [29,30,31,32,33,34,35,36,37,38,39,40,41,42,43,44,45,46,47,48,49,50]. Fortunately, the majority of the studies found a correlation between NAFLD and CKD with adjustment for these factors, as well as co-morbidities such as insulin resistance and metabolic syndrome [29,30,31,32,33,34,35,36,37,38,39,40,41,42,43,44,45,46,47,48,49,50].

While the prevalence of CKD in NAFLD appears to be substantial, studies that examine the incidence of CKD in NAFLD are not as robust [5]. The Valpolicella Heart Diabetes Study of 1760 patients with type 2 diabetes with preserved kidney function followed over a six-year period found an increased incidence of CKD in patients with NAFLD (HR 1.49; CI 95%, 1.1–2.2) independent of sex, age, blood pressure, duration of diabetes and smoking [31]. Additionally, a retrospective study on a cohort of 8329 non-diabetic, non-hypertensive men with normal kidney function revealed that NAFLD was associated with an increased incidence of CKD (HR 1.60; CI 95%, 1.3–2.0) over a three year period after adjustment for age, cholesterol, and other factors [31]. However, both of these studies also used ultrasound for the diagnosis of NAFLD [31,32]. Finally, none of these studies have used renal biopsy to examine the pathology of their CKD. In the future, randomized studies with larger cohorts of patients and longer follow-up and histologically confirmed fatty liver disease are needed to verify a causal relationship between NAFLD and CKD.

## 4. Mechanisms Linking NAFLD to CKD

**Figure 1:**
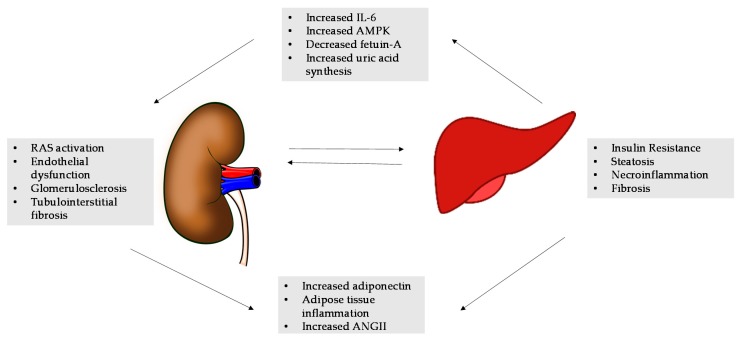
This figure demonstrates the various mechanisms associated with non-alcoholic fatty liver disease (NAFLD) and chronic kidney disease (CKD). The liver-kidney crosstalk in NAFLD includes altered renin-angiotensin system (RAS) and activated protein kinase (AMPK) activation, impaired antioxidant defense, and excessive dietary fructose intake, which affects renal injury through altered lipogenesis and inflammatory response. In turn, 8 the kidney reacts promoting further RAS activation, increased angiotensin II (ANGII) and uric acid production in a vicious cycle leading to fibrosis [20].

According to the Center for Disease Control (CDC), more than one-third of U.S. adults are obese [52]. This epidemic affects over 78 million people with co-morbidities of insulin resistance, diabetes, and atherosclerosis leading to an estimated annual medical cost of 147 billion dollars [52]. The liver is the key regulator of glucose and lipid metabolism as well as the main source of inflammatory elements thought to be involved in the development of cardiovascular and kidney disease [5]. It is known that obesity is an independent risk factor for CKD and it is associated with the development of proteinuria and pathologic findings of podocyte hypertrophy and focal segmental glomerular sclerosis even in the absence of diabetes and hypertension [53]. In addition, studies have shown that obesity as well as metabolic syndrome is a strong predictor of the development of NAFLD [54]. While the complex “crosstalk” among adipose tissue, the liver, and kidneys make it difficult to delineate the specific processes underlying NAFLD as a cause of CKD, it is not surprising that these diseases may be linked. Mounting evidence on liver-kidney interactions including; altered renin-angiotensin system (RAS) activation, impaired antioxidant defense, and damaged lipogenesis is currently emerging as a major area of research (Figure 1) [51]. Understanding these mechanisms may lead to modifiable risk factors and therapeutic targets for the prevention and treatment of NAFLD and CKD.

### 4.1. AMPK, Fetuin-A, and Adiponectin

The role of the energy sensor 5′-AMP activated protein kinase (AMPK) and its regulation of fetuin-A and adiponectin in liver and kidney fat cells is currently an area of investigation in animal models as well as human subjects [53]. Fetuin-A is a serum protein mediated through AMPK as an important promoter of insulin resistance found in both podocytes and hepatocytes [53]. Observations in fetuin-A null mice include resistance to weight gain when challenged with a high fat diet and increased insulin levels [55]. Similarly in humans, higher fetuin-A levels are associated with obesity and found in patients with NAFLD and CKD [55]. Adversely, adiponectin, which is regulated by fetuin A, is present in low levels with similar characteristics of elevated body mass index and hypertriglyceridemia [56]. Interestingly, therapeutic maneuvers including caloric restriction, exercise, and insulin sensitizing medications are associated with declines in levels of serum fetuin-A, increases in adiponectin levels, and stimulation of AMPK [53]. Although direct causation cannot be implied, it appears that increased caloric intake and adiposity initiates an inflammatory cascade through AMPK, fetuin-A, and adiponectin between fat cells in the liver and kidney leading to end-organ damage [53].

### 4.2. Renin-Angiotensin System (RAS) in NAFLD and CKD

The renin-angiotensin system (RAS) is also believed to play a key role in the pathogenesis of NAFLD and CKD. Adipocytes express all components of RAS and contribute up to 30% of circulating renin, angiotensin converting enzyme (ACE), and angiotensin II (AngII) [51]. The kidney and liver also express RAS constituents, and experimental studies support a role for both systemic and local activation of AngII in NAFLD and CKD. In the liver, AngII promotes insulin resistance, *de novo* lipogenesis, and pro-inflammatory cytokine production such as interleukin-6 (IL-6) and tumor growth factor-β (TGF-β) [51]. These processes are thought to trigger fibrogenesis contributing to the entire spectrum of histological changes seen with NASH [51]. In the kidney, RAS activation plays a key role in determining renal ectopic lipid deposition which is known to cause oxidative stress and inflammation through hemodynamic effects of glomerular efferent arteriole vasoconstriction leading to glomerulosclerosis [57]. In addition, a process known as the ACE2-Ang (1–7)-Mas receptor axes whose activity is known to oppose that of AngII has been shown in animal models to inhibit liver fibrosis [58]. The role of the RAS system in the liver and kidneys makes it a prime target for blockade in an attempt to attenuate fibrosis in NAFLD and CKD.

### 4.3. Fructose Metabolism in NAFLD and CKD

Based on the NHANESIII study, over 10% of Americans’ daily calories are from fructose and consumption in high fructose corn syrup (HFCS) has increased 8% over the last decade especially amongst adolescents [59]. Several observational studies have implicated HFCS in the incidence and severity of NAFLD and CKD [51]. Fructose acts independently of calorie excess by initiating fructose phosphorylation to fructose-1-phosphate by fructokinase in the liver, ultimately leading to the accumulation of uric acid [51]. Research investigations support that uric acid promotes the development and progression of NAFLD and CKD via hepatocyte ATP depletion, which causes enhanced hepatic and renal lipogenesis, mitochondrial ROS generation, endothelial dysfunction and pro-inflammatory cytokine secretion similar to overexpression of RAS [51]. Mouse models unable to metabolize fructose are protected from obesity, metabolic syndrome, and a reduction in fructose intake or uric acid production improved experimental NAFLD and CKD [60]. Also in a recent study of 341 adult NAFLD patients, investigators evaluated whether increased fructose consumption correlates with the development of NAFLD and found that after controlling for age, gender, BMI, and total calorie intake, increased daily fructose consumption was associated with lower steatosis grade and higher fibrosis stage in comparison to groups (*p* < 0.05) [61]. Finally, a meta-analysis examined four studies that assess the association between consumption of artificially sweetened soda verses regular soda and CKD and concluded the pooled risk reduction of CKD in patients consuming artificially sweetened soda was 1.33 (95% CI 0.82–2.15) [62]. Limitations in this study include its retrospective nature, which cannot imply causation as well as variability in types of soda consumed [62]. Future prospective studies on human subjects and limitations of fructose as well as reductions in uric acid levels in patients with NAFLD and CKD are necessary to confirm these hypotheses.

### 4.4. Impaired Oxidative Stress

As stated above increased oxidative stress is believed to play a key role in the pathogenesis of NAFLD and CKD. Nuclear erythroid related factor-2 (Nrf2), which is expressed ubiquitously in human tissues with its highest expression in the liver and kidney, upregulates the transcription of numerous antioxidant and detoxification enzymes by binding to their antioxidant response elements [63]. Experimental data support a key protective role for Nrf2 against NAFLD and CKD using wild-type and Nrf2-null mice fed a high fat diet. Their specimens were analyzed for pathology as well as for fatty acid content and revealed the wild-type mice had increased hepatic fat deposition without fibrosis while the Nrf2-null mice had significantly more hepatic steatosis and substantial inflammation [63]. Based on these results, several natural and artificial Nrf2 activators are being evaluated in the treatment of diabetic CKD patients in the “Bardoxolone ethyl and kidney function in CKD with type 2 diabetes (BEAM)” study and previously in the “Bardoxolone methyl evaluation in patients with chronic kidney disease and type 2 diabetes: the occurrence of renal events (BEACON)” trial [64,65]. Mechanisms linked to fibroblastic growth factor 21, gut microflora, and other proteins such as sirtuin-1 are also showing promise in the development of CKD in NAFLD [51].

## 5. Therapeutic Interventions in NAFLD and CKD

Based on the newer mechanisms discussed as well as aims at reducing insulin resistance, several therapeutic interventions for the treatment of NAFLD are currently under investigation. The mainstay of management of for NASH is lifestyle intervention, which includes diet and exercise with a 5%–10% weight reduction associated with improvement in hepatic steatosis [5]. While there are very few studies examining the use of medications and behavioral modification in both NAFLD and CKD, the shared cardiometabolic risk factors and underlying pathophysiology may make these therapies applicable to both diseases.

RAS blockade using angiotensin converting enzyme inhibitors (ACE-) and angiotensin receptor blockers (ARBs) has been studied in NAFLD and CKD. Limited data from 223 patients in three randomized controlled trials in NAFLD suggests that ARBs attenuate steatosis, insulin resistance, and inflammatory markers independent of reduction in blood pressure [51]. In addition, telmisartan which is an ARB with peroxisome proliferator activated receptor [PPAR]-γ-regulating activity was compared to the use of valsartan in the Fatty Liver Protection by Telmisartan (FANTASY Trial) and found to cause reduction in necroinflammation, NAFLD activity score, fibrosis stage in NASH, as well as microalbuminuria [66]. Not surprisingly, the use of these medications in CKD has been extensively evaluated and based on the Collaborative Study Group Trial and several others, the use of ACE- and ARBs in patients with CKD with proteinuria is now a level one recommendation by Kidney Disease Outcomes Quality Initiative (KDOQI) [67]. A recent cross-sectional study of 191 patients with CKD III, IV, ESRD, and renal transplant recipients (*n* = 68) treated with ACE- or ARBs for >1 year and examined liver stiffness with the use of TE and a CAP score to evaluate whether CKD patients receiving these medications have a lower frequency of NAFLD [68]. Investigators determined that CKD-NAFLD patients taking ACE-I or ARBs had lower degree of liver stiffness in comparison to the patients not on medications (*p* = 0.0005) [68]. However, there was no statistical significance in degree of fibrosis or grade of steatosis in the two groups based on CAP score [68].

Evidence from recent clinical trials suggests that insulin-sensitizing agents including thiazolidinediones (TZDs) such as pioglitazone are beneficial in the treatment of NAFLD. As stated above, pioglitazone is associated with a decline in levels of serum fetuin-A and concomitant increase in adiponectin levels resulting in decreased insulin resistance [53]. A recent meta-analysis using only liver biopsy studies, found that TZDs as well as pentoxyifylline, which has shown *in vitro* to inhibit proinflammatory cytokines as well as reduce fibrogenesis, are superior to placebo for improving steatosis and lobular inflammation [69]. This review also examined studies on obeticholic acid (OCA), a semi-synthetic bile acid analogue and vitamin E, both which have been used in the treatment of NAFLD and revealed improvement in ballooning degeneration and fibrosis in comparison to placebo [69]. While many of these studies have a small cohort of patients and the histological endpoints were not standardized, the American Association for the Study of Liver Disease (AASLD) published guidelines recommending the use of vitamin E and pioglitazone in non-diabetic adults with biopsy-proven NASH [69].

Pharmacologic treatments related to disordered cholesterol metabolism and insulin resistance including statins, fibrates, metformin, and glucagon-like peptide (GLP-1) analogues have shown potential benefit in adult patients with NAFLD and NASH [5]. However, the effects of these treatments are improvement in liver enzymes, decreased plasma glucose and weight loss without changes in histologic staging of the disease. There are three major post-hoc analysis reviewing the use of statins including the “Greek Atorvastatin and Coronary-Heart-Disease Evaluation” (GREACE), and “Incremental Decrease in End Points Through Aggressive Lipid Lowering” (IDEAL) trials that showed a significant reduction in cardiovascular disease events in patients with NAFLD/NASH [70,71]. Also, the GREACE study, revealed normal liver enzymes with the use of atorvastatin *versus* usual care in a three year follow-up period [51]. Therefore, it appears that the use of statins may also be safe in this patient population. Finally, lifestyle interventions including exercise, weight loss, and gastric bypass surgery will decrease hepatic fat content and inflammation, however, require significant effort and often financial burden on individual patients [5]. However, these may be worthwhile efforts in patients with early steatosis in order to prevent progression of to NAFLD with CKD. Novel therapies including translational approaches based on the mechanisms discussed, as well as more traditional methods need to be evaluated in large randomized controlled trials for their potential value in the treatment of both NAFLD and CKD.

## 6. Conclusions

Based on the data presented as well as several other ongoing trials, there is substantial evidence linking NAFLD to the development of CKD. It is clear that the mechanisms underlying these diseases are complexly inter-woven requiring additional investigation with animal and human models. Furthermore, prospective studies on NAFLD and CKD must include information on hepatic and renal histology. Preventative measures including lifestyle modification aiming toward weight loss and physical activity may be of benefit in both diseases. Furthermore, physician awareness for screening of CKD in NAFLD may lead to earlier detection and treatment of this disease leading to better outcomes in patients with liver steatosis as well as more advanced fibrosis requiring organ transplantation.

## Figures and Tables

**Table 1:** Non-alcoholic fatty liver disease (NAFLD) prognostic panels for fibrosis.

Reference	Test	Components	PPV%	NPV%
Rosenberg [17]	Original European Liver Fibrosis Panel	age, HA, TIMP1, PIIINP for score ≤1	80	98
Ratziu [18]	BAAT score	BMI ≥ 28 kg/m^2^ age ≥ 50 years, ALT ≥ 2 × ULN triglycerides ≥ 1.7 mmol/L	33	100
Ratziu [19]	Fibrotest	α2 macroglobulin, haptoglobin, GGT, Total bilirubin, apolipoprotein A1	54	90
Angulo [20]	NAFLD Fibrosis Score	age, hyperglycemia, BMI, platelet count, albumin, AAR	56	93
Harrison [21]	BARD	BMI ≥ 28 kg/m^2^, AAR ≥ 0.8, diabetes	43	96
Cales [22]	Fibrometer NAFLD	glucose, AST, ferritin, ALT, body weight, age	87.9	92.1
Shah [23]	FIB4 index	age, ALT, AST, platelet count	43	90
Sumida [24]	NAFIC score	serum ferritin (≥200 ng/mL for female, ≥300 ng/mL for male), fasting insulin ≥ 10	32	96
Younossi [25]	NAFLD Diagnostic Panel	diabetes, gender, BMI, triglycerides, apoptotic and necrotic CK18 fragments	57.7	85

This table demonstrates various prognostic panels for predicting the severity of fibrosis in NAFLD with respect to their positive predictive value (PPV) and negative predictive value (NPV) as determined by each study and its components. Abbreviations: BAAT=body mass index, aspart aminotransferase, age, triglycerides, HA = hyaluronic acid, TIMP1 = tissue inhibitor of matrix metalloproteinase, PIIINP = N-terminal propeptide of type III procollagen, BMI = body mass index, ALT = alanine aminotransferase, ULN = upper limit of normal , BARD = body mass index, aspart aminotransferase, alanine aminotransferase, diabetes, GGT = gamma-glutamyl transpeptidase, AAR = aspart aminotransferase alanine aminotransferase ratio, AST = aspart transaminase, CK18 = creatinine kinase 18.

**Table 2:** Principal retrospective studies of the association between nonalcoholic fatty liver disease and the prevalence of chronic kidney disease (CKD).

Study	Characteristics	CKD Diagnosis and Prevalence	Liver Disease Diagnosis and Prevalence	Risk Factors Adjusted in Analysis
Targher, 2008 [29]	Outpatient; *n* = 103; HTN 63%	eGFR < 60 mL/min/1.73 m^2^ (CKD-EPI) and/or overt proteinuria; 15%	Ultrasound; 48%	Age, sex, BMI, waist circumference, HTN, alcohol consumption, diabetes duration, HbA1c, LDL cholesterol, Tg
Campos, 2008 [30]	Hospital; *n* = 197; HTN 56%, DM 26%	eGFR < 60 mL/min/1.73 m^2^ (CKD-EPI); 10%	Liver biopsy: NAFLD 63%, NASH 32%	Age, gender, BMI, waist circumference, HTN
Chang, 2008 [31]	Population; *n* = 8329; DM 0%, HTN 0%, metabolic syndrome 6%	eGFR < 60 mL/min/1.73 m^2^ (MDRD) or morning proteinuria >1+; 4%	Ultrasound; 73%	Age, eGFR, dyslipidemia, BMI, CRP, sys BP
Targher, 2008 [32]	Population; *n* = 1760; DM 100%, HTN 65%, metabolic syndrome 55%	eGFR < 60 mL/min/1.73 m^2^ (MDRD) or ACR = 300 mg/g; 31%	Ultrasound; 30%	Age, gender, BMI, waist circumference, BP, LDL-C, Tg, smoking, DM duration, medications
Targher, 2010 [33]	Outpatient; *n* = 202 adults; HTN 35%, DM 0%	eGFR < 60 mL/min/1.73 m^2^ and/or ACR ≥ 30 mg/g; 37.8%	Ultrasound	Age, sex, BMI, systolic BP, alcohol consumption, diabetes duration, HbA1c, Tg, medication use
Targher, 2010 [34]	Hospital; *n* = 160; DM 6%, HTN 60%, metabolic syndrome 29%	eGFR < 60 mL/min/1.73 m^2^ (CKD-EPI) or ACR = 30 mg/g; 14%	Biopsy; NASH 100%	Age, sex, BMI, waist circumference, smoking, systolic BP, insulin resistance
Yilmaz, 2010 [35]	Hospital; *n* = 87; DM 0%, HTN 30%, metabolic syndrome 27%	eGFR < 60 mL/min/1.73 m^2^ (CKD-EPI) or ACE 30–300 mg/d; 16%	Biopsy; NAFLD 100%, NASH 67%	Age, gender, BMI, waist circumference, BP, lipids, smoking, insulin resistance, metabolic syndrome
Soderberg, 2010 [36]	Hospital; *n* = 125; DM 24%, HTN 37%, metabolic syndrome 31%	eGFR < 60 mL/min/1.73 m^2^ (CKD-EPI); 27%	Biopsy; NAFLD 67%, NASH 33%	Age, BMI, HTN, smoking, DM, metabolic syndrome
Wong 2010 [37]	Hospital; *n* = 51; DM 50%, HTN 37%, metabolic syndrome 65%	eGFR < 60 mL/min/1.73 m^2^ (CKD-EPI) or ACR > 30mg/g; 8%	Biopsy; NAFLD 100%, NASH 33%	Age, BMI, DM, HTN, waist circumference, metabolic syndrome, smoking
Lau 2010 [38]	Population; *n* = 2858; DM 8.9%, HTN 47%; metabolic syndrome 24%	eGFR < 60 mL/min/1.73 m^2^ (CKD-EPI) or ACR > 30 mg/g; 8%	Ultrasound; 30%	Age, BMI, metabolic syndrome, HTN, dyslipidemia, smoking
Yasui 2011 [39]	Hospital; *n* = 169; DM 31%, HTN 34%, metabolic syndrome 30%	eGFR < 60 mL/min/1.73 m^2^ (CKD-EPI) or am proteinuria 1+; 14%	Biopsy; NAFLD 100%, NASH 53%	BMI, HTN, waist circumference, dyslipidemia, smoking, DM
Machado 2012 [40]	Hospital; *n* = 148; HTN 67%	eGFR < 60 mL/min/1.73 m^2^; 8%	Biopsy; NAFLD 100%	Age, sex, HTN, DM, dyslipidemia
Targher 2012 [41]	Hospital; *n* = 343; DM 100%, HTN 43%, metabolic syndrome 46%	eGFR < 60 mL/min/1.73 m^2^ (MDRD) or ACR > 30 mg/g; 40%	Ultrasound 53%	Age, gender, BMI, family history, systolic BP, dyslipidemia, smoking DM, medications, microalbuminuria
Sirota 2012 [42]	Population; *n* = 11469; HTN 24%	eGFR < 60 mL/min/1.73 m^2^ and/or ACR > 30 mg/g; 42%	Ultrasound	Age, sex, race, HTN, diabetes, waist circumference, dyslipidemia, insulin resistance
Armstrong 2012 [43]	Population; *n* = 146; DM 0%, HTN 36%	eGFR < 60 mL/min/1.73 m^2^ (CKD-EPI); 25%	Ultrasound; 50%	BMI, HTN
Musso 2012 [44]	Hospital; *n* = 80; DM 0%, HTN 52%, metabolic syndrome 31%	eGFR < 60 mL/min/1.73 m^2^ (CKD-EPI) or ACR > 30 mg/d; 20%	Biopsy; NAFLD 50%, NASH 20%	Age, gender, BMI, waist circumference, HTN, smoking, metabolic syndrome
Francque 2012 [45]	Hospital; *n* = 230; DM 0%, HTN 50%, metabolic syndrome 47%	eGFR < 60 mL/min/1.73 m^2^ (CKD-EPI) or proteinuria > 300 mg/d; 9%	Biopsy; NAFLD 100%, NASH 52%	Age, BMI, HTN, waist circumference, smoking, metabolic syndrome
Casoinic 2012 [46]	Hospital; *n* = 145; DM 100%; HTN 55%; metabolic syndrome 80%	eGFR < 60 mL/min/1.73 m^2^ (CKD-EPI) or ACE 30–300 mg/g; 10%	Ultrasound; 51%	Age, gender, CRP
Xia 2012 [47]	Population; *n* = 1141; DM 0%, HTN 38%, metabolic syndrome 32%	eGFR < 60 mL/min/1.73 m^2^ (mDRD) or ACR > 30 mg/g; 12%	Ultrasound; 41%	Age, BMI, smoking, HTN, metabolic syndrome, uric acid
Kim 2013 [48]	Hospital; *n* = 96; DM 100%, HTN 66%, metabolic syndrome 56%	eGFR < 60 mL/min/1.73 m^2^ (MDRD) or proteinuria > 1+ am; 25%	Biopsy: NAFLD 100%, NASH 56%	Age, BMI, HTN, waist circumference, smoking, metabolic syndrome, dyslipidemia
Angulo 2013 [49]	Hospital; *n* = 191; DM 17%, HTN 32%, metabolic syndrome 25%	eGFR < 60 mL/min/1.73 m^2^ (CKD-EPI) or am proteinuria >1+; 18%	Biopsy	Age, BMI, DM, HTN, smoking, dyslipidemia, metabolic syndrome
El Azeem 2013 [50]	Population; *n* = 747; DM 57%, HTN 32%, metabolic syndrome 67%	eGFR < 60 mL/min/1.73 m^2^ (MDRD) or ACE > 30 mg/g; 29%	Ultrasound 35%	Age, BMI, HTN, dyslipidemia, smoking, metabolic syndrome

This table represents the major retrospective studies linking the prevalence of CKD in NAFLD. The data is organized chronologically and include the cohort, definition of CKD and NAFLD with prevalence as well as adjustment variables. Studies using liver enzymes for the diagnosis of NAFLD or survey data were not included in this review. Abbreviations: HTN = hypertension, DM = diabetes mellitus, eGFR = estimated glomerular filtration rate, CKD-EPI = chronic kidney disease epidemiology collaboration, MDRD = modification of diet in renal disease, BMI=body mass index, HbA1C = hemogloblin A1C %, LDL = low density lipoprotein, Tg = triglyceride, BP = blood pressure, CRP = c-reactive protein.

## Abbreviations

DM	diabetes mellitus
HTN	hypertension
Tg	triglycerides
A1C%	hemoglobin A1C
eGFR	estimated glomerular filtration rate
MDRD	Modification of Diet in Renal Disease
CKD-EPI	Chronic Kidney Disease Epidemiology Collaboration
CRP	C-reactive protein
LFTs	liver function tests
HR	hazard ration
CI	confidence interval
